# Anger tendency may be associated with duration of illness in panic disorder

**DOI:** 10.1186/s13030-015-0035-3

**Published:** 2015-03-01

**Authors:** Nagisa Sugaya, Eiji Yoshida, Shin Yasuda, Mamoru Tochigi, Kunio Takei, Takeshi Otowa, Tadashi Umekage, Yoshiaki Konishi, Yuji Sakano, Shinobu Nomura, Yuji Okazaki, Hisanobu Kaiya, Hisashi Tanii, Tsukasa Sasaki

**Affiliations:** Department of Epidemiology and Public Health, Graduate School of Medicine, Yokohama City University, 3-9 Fukuura, Kanazawa-ku, Yokohama, Kanagawa 236-0004 Japan; Outpatient Clinic for Anxiety Disorders, Akasaka Clinic, 3-9-18 Akasaka, Minato-ku, Tokyo 107-0052 Japan; Kinkou Hospital, Kanagawa Psychiatric Center, 2-5-1, Serigaya, Kounan-ku, Yokohama, Kanagawa 233-0006 Japan; Neural Plasticity Project, Tokyo Metropolitan Institute of Medical Science, 2-1-6 Kamikitazawa, Setagaya-ku, Tokyo 156-8506 Japan; Department of Neuropsychiatry, Graduate School of Medicine, The University of Tokyo, 7-3-1 Hongo, Bunkyo, Tokyo 113-8655 Japan; Health Service Center, The University of Tokyo, 7-3-1 Hongo, Bunkyo-ku, Tokyo 113-8654 Japan; Office for Mental Health Support, The University of Tokyo, 7-3-1 Hongo, Bunkyo-ku, Tokyo 113-8654 Japan; Department of Psychiatry, Mie University Graduate School of Medicine, 2-174 Edobashi, Tsu, Mie 514-8507 Japan; School of Psychological Science, Health Sciences University of Hokkaido, 2-5 Ainosato, Kita-ku,, Sapporo, Hokkaido 002-8072 Japan; Faculty of Human Sciences, Waseda University, 2-579-15 Mikajima, Tokorozawa, Saitama 359-1192 Japan; Department of Psychiatry, Koseikai Michinoo Hospital, 1-1 Nijigaoka-machi, Nagasaki City, Nagasaki 852-8055 Japan; Research Center for Panic Disorder, Nagoya Mental Clinic, 1-16 Tsubaki-cho, Nakamura-ku, Nagoya, Aichi 453-0015 Japan; Graduate School of Education, The University of Tokyo, 7-3-1 Hongo, Bunkyo-ku, Tokyo 113-0033 Japan

**Keywords:** Panic disorder, Illness duration, Anger, Personality

## Abstract

**Background:**

Several studies have reported an increased tendency towards anger in patients with panic disorder (PD). If this propensity for anger arises from the pathological process of PD, it may be associated with the duration of the illness. The present study therefore examined the relationship between duration of PD and the personality tendency to experience anger in PD patients.

**Methods:**

Participants were 413 patients (132 men and 281 women; age = 38.7 years) with PD. Diagnoses were confirmed using the Mini-International Neuropsychiatric Interview. Illness duration ranged from less than a year to 51 years. After participants completed the Revised NEO Personality Inventory, we examined the association between illness duration and the Angry Hostility and Impulsiveness subscale scores. In the analysis, participants were divided into two groups by duration of illness (long group, n = 186 and short group, n = 200) using the median value (9 years) as a cut-off because of the skewed distribution of the duration. Patients with an illness duration of 9 years (n = 27) were excluded from the comparison.

**Results:**

The duration of illness was significantly correlated with the Angry Hostility score (*p* = 0.002) after controlling for age. Scores were significantly higher in the long group than in the short group (*p* = 0.04). No significant association was observed between Impulsiveness scores and duration of illness.

**Conclusion:**

The present study suggests that longer PD duration is related to a stronger tendency to experience anger.

## Introduction

A number of studies have reported an association between panic disorder (PD) and the experience or expression of anger. Patients with PD may have a significantly greater propensity to experience and express anger through aggressive behavior than do healthy controls [1]. Additionally, extreme expressions of anger, or “anger attacks” are more commonly found in patients with PD [2]. An anger attack is defined as a sudden spell of intense anger that is inappropriate to the situation. Individuals who experience anger attacks may yell or verbally abuse others, throw or damage objects, or feel out of control [3]. Panic attacks and anger attacks are both associated with a variety of depressive and anxiety disorders, including obsessive-compulsive disorder, generalized anxiety disorder, panic disorder, and so forth [4,5].

The underlying mechanism of the association between PD and anger remains unknown, but duration of illness may explain the propensity for anger in PD. Patients may become more irritable as a long-term effect of the illness, possibly through a change of personality. Indeed, a previous retrospective study on personality disorders reported that the duration of PD was a statistically significant predictor of cluster B and C personality disorders, suggesting that long-lasting PD might be related with the development of personality disorders [6]. Cluster B personality disorders include antisocial and borderline personality disorders, which are closely related with a propensity for anger, aggressiveness, and impulsiveness. From our clinical observations, we have often found that patients with long-lasting PD are more prone to experience and impulsively express anger. Although personality may be considered hard-wired, a previous study reported that personality can change throughout the life course [7]. Thus, long-lasting PD might give rise to more irritable and impulsive personalities; therefore, patients with long-lasting PD might have a greater propensity to experience anger and express it through aggressive behavior.

However, few studies have investigated the association between duration of PD and the propensity for anger. In the present study, we examined this association, hypothesizing that longer duration of illness is related to an increased propensity to experience and impulsively express anger, as reflected in subjects’ personality characteristics.

## Methods

### Participants

A total of 472 outpatients with PD, who met the DSM-IV criteria for the disorder [8] and were treated at one of two psychiatric clinics specializing in anxiety disorders, were consecutively selected and participated in this study between May 2004 and April 2010. The clinics were located in Tokyo and Nagoya, Japan. Diagnoses of PD and PD with agoraphobia were confirmed by interviews using the Mini-International Neuropsychiatric Interview (MINI) and review of clinical charts.

Valid data were obtained from 413 patients with PD, including 132 men and 281 women (attrition rate = 12.5%). The mean age (SD) of participants was 38.7 (10.5) years. The mean age of PD onset was 28.3 (10.0) years, and mean duration of PD was 10.4 (8.4) years. Agoraphobia was present in 57.6% of the 413 patients according to the interviews using MINI.

### Measures

#### The mini-international neuropsychiatric interview

The MINI is a brief structured interview for assessing major DSM-IV Axis I psychiatric disorders [9]. We used the MINI to confirm the diagnosis of PD and the presences of agoraphobia, other anxiety disorders (social anxiety disorder (SAD), obsessive-compulsive disorder (OCD), generalized anxiety disorder (GAD), posttraumatic stress disorder (PTSD)), and current affective disorders (major depressive episode, manic episode, and hypomanic episode) in the participants of this study.

#### Angry hostility and impulsiveness subscales in the revised NEO personality inventory

The Revised NEO Personality Inventory (NEO PI-R) [10-12] is a self-report questionnaire for measuring the five major domains of personality: Neuroticism, Extraversion, Openness, Agreeableness, and Conscientiousness. Neuroticism reflects personality characteristics that make people prone to psychological distress; it contains six facets: Anxiety, Angry Hostility, Depression, Self-Consciousness, Impulsiveness, and Vulnerability. We used two subscales of the Neuroticism domain: Angry Hostility and Impulsiveness (internal consistencies are 0.75 and 0.74, respectively [12]). The Angry Hostility scale assesses the tendency to experience anger and related states, such as frustration and bitterness. The Impulsiveness scale assesses the tendency to act on urges and to have difficulty controlling anger or cravings. We did not investigate other subscales of the Neuroticism scale or Extraversion, Openness, Agreeableness, or Conscientiousness scales in the present study.

### Procedure

Members of our research team (consisting of psychiatrists and clinical psychologists) conducted structured interviews using the MINI to confirm the diagnosis of PD and the presence or absence of agoraphobia in each participant. Diagnosis of PD was additionally confirmed by a review of each participant's clinical records. Participants then completed the NEO PI-R.

## Ethical considerations

The researchers provided detailed explanations of the purpose of this study, the contents of each test, and the protection of personal and other information. All patients with PD who agreed to participate in this study provided their informed consent. The study was approved by the Ethical Committee of the Graduate School of Medicine, University of Tokyo.

### Statistical analysis

Data analysis was performed using SPSS 20.0 software (SPSS Japan Inc.). The Kolmogorov–Smirnov test did not show a normal distribution of PD duration in the participants. Thus, participants were divided into long and short PD duration groups according to the median PD duration (9 years); that is, participants in the long PD duration group had PD that had lasted for over 9 years, while those in the short PD duration group had PD that had lasted less than 9 years. Patients with an illness duration of 9 years (*n* = 27) were excluded from the comparison between the long and short groups. Comparisons of scores for Angry Hostility and Impulsiveness by sex, as well as usage of benzodiazepines and the presence of agoraphobia, other anxiety disorders, and affective disorders were analyzed using a *t*-test. Correlational analysis was performed using Pearson's correlation coefficient. The following correlations were analyzed: Angry Hostility and age, Impulsiveness and age, and the dosages of selective serotonin reuptake inhibitors (SSRIs) prescribed, including fluvoxamine, paroxetine, and milnacipran or the imipramine-equivalents of antidepressants, including imipramine, SSRIs, and sulpiride. We compared the mean ages and the prescribed dosages of SSRIs and imipramine-equivalents between the long and short groups using a *t*-test. Comparisons of sex ratio, the usage of benzodiazepines, and the prevalence of agoraphobia, other anxiety disorders, and affective disorders between the groups were performed using a chi-square test. These comparisons were analyzed to determine if controls for those variables were necessary in the statistical examination of the relationship between duration of PD and the Angry Hostility and Impulsivity scores. When a variable is significantly related to the duration of illness as well as to the personality score (as measured by the NEO PI-R), the variable should be controlled for as a covariate. Comparisons of the subscale scores of the NEO PI-R between the groups were made using an analysis of covariance (ANCOVA). For the ANCOVA, we selected only variables showing significant differences between the groups. Partial correlation analysis was then performed for all participants (N = 413) to confirm any relationships between duration of PD and the subscale scores of the NEO PI-R. The significance level was set at *p* < 0.05.

## Results

**Figure 1:**
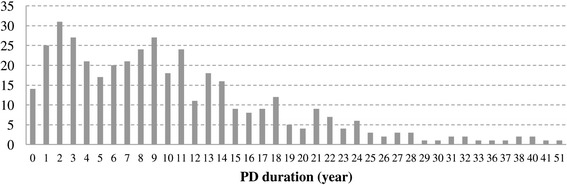
**Distribution of panic disorder (PD) duration.**

**Figure 2:**
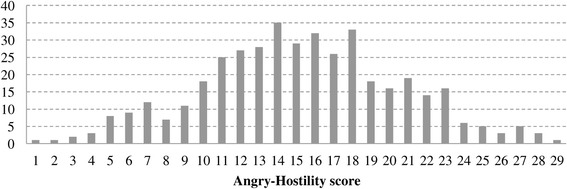
**Distribution of Angry Hostility score.**

**Figure 3:**
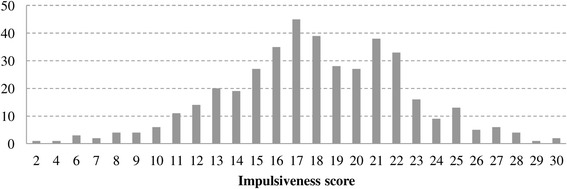
**Distribution of impulsiveness score.**

According to our group definitions, 186 patients (58 men and 128 women, 42.4 (10.0) years) were assigned to the long PD duration group (long group) and 200 patients (66 men and 134 women, 34.7 (9.5) years) to the short PD duration group (short group). Figures 1, 2, and 3 show the distributions of PD duration, Angry Hostility scores, and Impulsiveness scores, respectively. The long group had a significantly longer duration of illness than the short group (*t* (224.7) = 23.6, *p* < 0.0001).

We confirmed the presence of a relationship between propensity to anger and age, sex, prescription medications, and the prevalence of comorbid disorders. A significant correlation was revealed between the Angry Hostility score and age (*r* = −0.17, *p* = 0.001). With the Impulsiveness score, the following variables were significantly associated: age (*r* = −0.21, *p* < 0.001), imipramine-equivalents (*r* = 0.15, *p* = 0.003), and prescribed dosage of fluvoxamine (*r* = 0.10, *p* = 0.046). PD patients with the following comorbid disorders and conditions had significantly higher scores for Angry Hostility and Impulsiveness: Generalized Anxiety Disorder (GAD) (*t*(411) = 3.1, *p* = 0.002, and *t*(411) = 3.4, *p* = 0.001, respectively), major depressive episode (*t*(411) = 3.3, *p* = 0.001, and *t*(411) = 2.8, *p* = 0.005, respectively), manic episode (*t*(411) = 4.7, *p* < 0.001, and *t*(411) = 2.8, *p* = 0.005, respectively) and hypomanic episode (*t*(411) = 2.6, *p* = 0.009, and *t*(411) = 3.1, *p* = 0.002, respectively). PD patients with agoraphobia and PTSD scored higher on the Impulsiveness scale (*t*(411) = 4.3, *p* < 0.001 and *t*(411) = 2.2, *p* = 0.03, respectively); scores for Angry Hostility, however, were not significantly different. PD patients with Social Anxiety Disorder (SAD) scored higher on the Angry Hostility scale (*t*(411) = 2.0, *p* = 0.04) than those without SAD, but showed no differences in regard to Impulsivity. Among the variables significantly associated with Angry Hostility and Impulsivity scores, age was significantly associated with duration of illness. In the long duration group, participants were significantly older than participants in the short duration group (*t*(384) = 7.7, *p* < 0.0001); other variables were not associated with duration of illness (Table 1). We, therefore, controlled for age as a continuous covariate when we tested for a relationship between duration of illness and Angry Hostility, and duration of illness and Impulsivity; other variables were not adjusted for.

**Table 1:** **Sex ratio, age, and clinical characteristics of the participants**

	**Total**	**Long PD duration**	**Short PD duration**
N (Male/Female)	413 (132/281)	186 (58/128)	200 (66/134)
Age (mean (SD))	38.7 (10.5)	42.4 (10.0)	34.7 (9.7)
PD duration (mean (SD))	10.4 (8.4)	17.5 (7.4)	4.0 (2.5)
Fluvoxamine (mean (SD/range)) [mg]	23.1 (36.1/0-300)	23.7 (39.8/0-300)	23.1 (33.9/0-150)
(N (%) of users)	172 (42.2%)	75 (41.0%)	85 (42.9%)
Paroxetine (mean (SD/range)) [mg]	7.8 (11.6/0-40)	7.1 (11.5/0-40)	8.6 (11.8/0-40)
(N (%) of users)	155 (38.0%)	63 (34.4%)	83 (41.9%)
Milnacipran (mean (SD/range)) [mg]	2.2 (11.3/0-100)	3.1 (13.4/0-100)	1.4 (9.1/0-100)
(N (%) of users)	19 (4.7%)	12 (6.6%)	6 (3.0%)
Benzodiazepine (N (%) of users)	386 (94.6%)	178 (97.3%)	183 (92.4%)
Imipramine-equivalents (mean (SD))	73.1 (60.0)	75.6 (65.5)	71.7 (56.0)
Agoraphobia (N (%))	238 (57.6%)	112 (60.2%)	108 (54.0%)
SAD (N (%))	24 (5.8%)	13 (7.0%)	9 (4.5%)
OCD (N (%))	19 (4.6%)	7 (3.8%)	10 (5.0%)
GAD (N (%))	19 (4.6%)	8 (4.3%)	11 (5.5%)
PTSD (N (%))	9 (2.2%)	4 (2.2%)	5 (2.5%)
Major depressive episode (N (%))	51 (12.3%)	27 (14.5%)	20 (10.0%)
Manic episode (N (%))	12 (2.9%)	5 (2.7%)	6 (3.0%)
Hypomanic episode (N (%))	24 (5.8%)	9 (4.8%)	14 (7.0%)

SD: standard deviation.

PD: panic disorder.

SAD: social anxiety disorder.

OCD: obsessive-compulsive disorder.

GAD: generalized anxiety disorder.

PTSD: posttraumatic stress disorder.

PD duration and age were significantly longer and higher in the long group than in the short group.

Sex ratio, prescriptions, and percentages of participants with agoraphobia or other comorbid disorders showed no significant differences between the long and short groups.

The data for prescription strength and usage was obtained from 408 participants (long duration group = 183, short duration group = 198).

**Figure 4:**
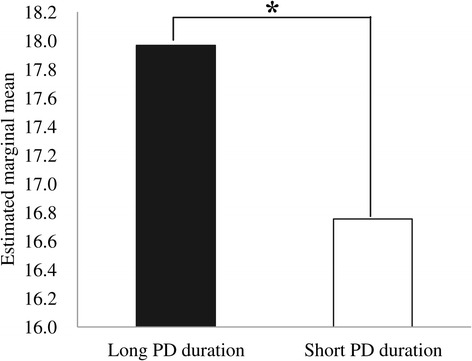
**Comparison of Angry Hostility score between the long and short panic disorder (PD) duration groups, after controlling for age** * **p = 0.04 (analysis of covariance) Angry Hostility scores were significantly higher in the long PD duration group than in the short group.**

PD duration (among all participants, N = 413) was significantly correlated with Angry Hostility scores (*r* = 0.15, *p* = 0.002) after controlling for age, but not with Impulsiveness scores (*r* = 0.07, *p* = 0.18). Furthermore, Angry Hostility scores were significantly higher in the long group than in the short group (*F* (1, 383) = 4.4, *p* = 0.04, Figure 4), while Impulsiveness scores did not significantly differ between the groups (*F* (1, 383) = 2.4, *p* = 0.12).

## Discussion

The results of the present study partly supported our hypothesis. Angry Hostility scores were significantly associated with PD duration, but the association between Impulsiveness scores and PD duration was not significant. The results suggest that longer PD duration may be associated with a stronger tendency to experience anger, but not impulsiveness. Angry Hostility scores were significantly higher in the longer duration group than the shorter duration group after controlling for age. Although the correlation between Angry Hostility scores and PD duration was statistically significant (*p* = 0.002), the correlation was weak (*r* = 0.15). Thus, we must carefully evaluate the clinical implications of these findings. A number of factors may play a role in the development of anger tendencies in PD patients, and PD duration might be one of them.

The mechanism underlying the association between PD duration and propensity for anger remains unclear. A previous study reported that longer PD duration correlates with smaller pituitary volume, which could be caused by chronic activation of the hypothalamic–pituitary–adrenal axis [13]. The pituitary gland secretes oxytocin, which has been found to reduce individuals’ feelings of anger towards people who have betrayed them, likely by strengthening these individuals’ understanding and acceptance of the reason that they had been betrayed [14]. Moreover, a previous study observed amygdala changes in PD [15], which could be related with altered expression of fear and aggression. Thus, long-duration PD patients could become more susceptible to hostility through activation of the amygdala. Biological mechanisms, including pituitary volume and oxytocin levels, might warrant investigation of mechanisms underlying the association between PD duration and propensity for anger in future research. In addition to biological mechanisms, environmental factors could underlie the association between PD duration and propensity for anger. Persistent symptoms of PD might disturb daily life among patients with PD, and their disabling condition could possibly cause chronic frustration and heightened irritability. This may be an interesting issue for future studies.

Another explanation may be comorbidity of bipolar disorder. Studies have reported that PD patients may have a high comorbid rate of bipolar disorder [16-18]. The prevalence of manic and hypomanic episodes in the present PD subjects (2.9% and 5.8%, respectively) was higher than the lifetime prevalence of manic and hypomanic episodes in the general Japanese population (0.5% and 0.2%, respectively) [19]. Patients with bipolar disorder may have an increased tendency to experience anger during manic, hypomanic, or other phases of the disorder. Furthermore, if PD duration is longer, the opportunity to experience anger during such phases is elevated. This could lead to the association between anger and PD duration. However, in our previous study, we found no significant difference in PD duration between patients with and without bipolar disorder among the same sample as the present study [18]. The present study did not show a relationship between PD duration and the presence of manic or hypomanic episodes, either. Our studies therefore do not support high comorbidity of bipolar disorder as a major cause of the association between increased anger and PD duration.

Another possible explanation for the association is that the illness could be more prolonged when patients have a stronger propensity for anger, which opposes the previous two explanations. In clinical expressions, improvement of emotional difficulties in patients with a stronger propensity for anger could be more challenging and require longer treatment. This is supported by a previous study that suggested a relationship between aggression and affective dependence [20]. Detailed clinical studies may be required to test this hypothetical explanation.

In future research, it may be necessary to conduct international comparisons on the association between PD duration and Angry Hostility. A cross-cultural study [21] shows that Japanese people experience less anger for shorter periods than do individuals from the US. Japanese culture appreciates conflict avoidance to a greater extent [22], and restricting anger helps maintain good social networks [23,24]. Thus, a stronger tendency towards suppressing anger among Japanese people might have influenced our findings.

The relationship between a propensity to become angry and illness duration may not be unique to PD. Previous research found that the illness duration of major depressive episodes was longer in patients who exhibited overt irritability and anger [25]. In the future, we will need to compare the relationship between a propensity to become angry and illness duration between PD and other psychiatric disorders.

We must acknowledge the following limitations in the present study. First, this was a retrospective study. All possible mediators and confounders may not have been examined. In a future prospective study, we should investigate whether or not a greater propensity to become angry exists at or before the onset of the illness in PD patients with longer illness duration or if it develops during the course of the illness. Second, we did not collect data on factors that may mediate the association between anger and PD duration. Brain structure or function (e.g., pituitary) and neurotransmitters could be possible factors that mediate the association. Third, we used a self-reported questionnaire to assess propensity to experience or impulsively express anger. Future research using objective measurements may help to obtain more conclusive results. Fourth, we did not make a distinction between anger toward others and anger toward oneself in this study. In future research, we need to investigate how PD duration relates to anger toward others and to anger toward the self.

## Conclusion

Patients with a long duration of PD showed a greater tendency towards anger than did patients with a short duration. The relationship between PD duration and tendency towards anger may be worthy of further studies, including a prospective study.

## Abbreviations

PD: Panic disorder
MINI: Mini-International Neuropsychiatric Interview
SAD: Social anxiety disorder
OCD: Obsessive-compulsive disorder
GAD: Generalized anxiety disorder
PTSD: Posttraumatic stress disorder
NEO PI-R: Revised NEO Personality Inventory
SSRI: Selective serotonin reuptake inhibitor
ANCOVA: Analysis of covariance
